# Preclinical and Clinical Feasibility Studies as the First Step Before Forthcoming Intravesical Instillation of [^211^At]At-anti-CA-IX Antibody (ATO-101™) Study in Patients with Non-Muscle-Invasive Bladder Cancer Unresponsive to Standard of Care

**DOI:** 10.3390/cancers17071190

**Published:** 2025-03-31

**Authors:** Caroline Rousseau, Pierre Baumgartner, Marie-Françoise Heymann, Manon Taupin, Maïwenn Geffroy, Jean-François Chatal, Gaëlle Gautier, Nadia Allam, Joëlle Gaschet, Romain Eychenne, François Guérard, Jean-François Gestin, Nicolas Varmenot, Michel Chérel

**Affiliations:** 1 Institut de Cancérologie de l’Ouest, F-44800 Saint-Herblain, France; pierre.baumgartner@ico.unicancer.fr (P.B.); marie-francoise.heymann@ico.unicancer.fr (M.-F.H.); manon.taupin@ico.unicancer.fr (M.T.); maiwenn.geffroy@ico.unicancer.fr (M.G.); nadia.allam@ico.unicancer.fr (N.A.); joelle.gaschet@ico.unicancer.fr (J.G.); jean-francois.gestin@univ-nantes.fr (J.-F.G.); nicolas.varmenot@ico.unicancer.fr (N.V.); michel.cherel@univ-nantes.fr (M.C.); 2 Nantes Université, INSERM, CNRS, CRCI2NA, Univ Angers, F-44000 Nantes, France; romain.eychenne@univ-nantes.fr (R.E.); francois.guerard@univ-nantes.fr (F.G.); 3 Research Pathology Platform, Tumor Heterogeneity and Precision Medicine, F-44800 Saint-Herblain, France; 4 Research Pathology Platform, F-44800 Saint-Herblain, France; 5 Atonco, Pôle Bio-Ouest Laennec, Rue du Moulin de la Rousselière, F-44800 Saint-Herblain, France; jf.chatal@atonco-pharma.com; 6 Chelatec, 1 Rue Aronnax, F-44817 Saint-Herblain, France; gaelle.gautier@chelatec.com; 7 Groupement d’Intérêt Public ARRONAX, 1 Rue Aronnax, F-44817 Saint-Herblain, France

**Keywords:** non-muscle-invasive bladder cancer, carbonic anhydrase IX, [^211^At]At, radioimmunotherapy, [^89^Zr]Zr-girentuximab, PET/CT, dosimetry

## Abstract

Introduction: Recently, alpha-emitting radionuclides like astatine-211 have offered promising results in clinical development. Non-muscle-invasive bladder cancer (NMIBC) presents a need for novel therapies. One promising approach is radioimmunotherapy targeting Carbonic Anhydrase IX (CA-IX), which is supported by preclinical and clinical evidence. The aim of our preclinical and clinical studies was to evaluate the [^211^At]At-anti-CA-IX antibody (ATO-101™) for future use in NMIBC patient care. Methods: The anti-CA-IX antibody, girentuximab (TLX250), was labeled with lutetium-177 and astatine-211 for in vitro studies. Affinity constant measurements of [^211^At]At-girentuximab in RT-112 cells were taken, and toxicity evaluations were conducted in vitro and in healthy mice. Additionally, a clinical proof-of-concept study, PERTINENCE, that used [^89^Zr]Zr-girentuximab for PET/CT imaging in bladder cancer patients was conducted. Results: The measurement of the affinity constant of [^211^At]At-girentuximab in RT112 cells revealed high binding affinity and significant cytotoxicity compared to [177Lu]Lu-girentuximab. Biodistribution studies in healthy mice indicated low systemic radioactivity uptake, and a bladder post-instillation examination showed no abnormalities in bladder mucosa, suggesting safety. In the PERTINENCE study, which involved patients with NMIBC tumors expressing CA-IX, [^89^Zr]Zr-girentuximab PET/CT showed no extravesical leakage. Wall bladder uptake spots correlated with recurrence or inflammatory reaction. A dosimetric study suggested the potential efficacy and favorable safety profile of intravesical alpha therapy with the [^211^At]At-anti-CA-IX antibody (ATO-101™) in NMIBC treatment. Conclusions: Preclinical and clinical data demonstrate the promising therapeutic role of ^211^At-targeted alpha agents in NMIBC, and the [^211^At]At-anti-CA-IX antibody (ATO-101™) could fulfill this role. A phase I FIH clinical trial is in preparation, and results are expected within the next years.

## Simple Summary

Non-muscle-invasive bladder cancer (NMIBC) presents a need for novel therapies. One promising approach is radioimmunotherapy targeting Carbonic Anhydrase IX (CA-IX) with a particular interest in alpha-emitting radionuclides like astatine-211. The aim of our preclinical and clinical studies was to assess the potential of [^211^At]At-anti-CA-IX antibody (ATO-101™) in NMIBC patient care. The measurement of the affinity constant of [^211^At]At-girentuximab in RT112 cells revealed high binding affinity and significant cytotoxicity compared to [177Lu]Lu-girentuximab. Biodistribution studies with [^211^At]At-girentuximab in healthy mice indicated low systemic radioactivity uptake, and a bladder post-instillation examination showed no abnormalities, suggesting safety. In the PERTINENCE study, patient [^89^Zr]Zr-girentuximab PET/CT showed no extravesical leakage. Wall bladder uptake spots correlated with recurrence or inflammatory reaction. A dosimetric study suggested the potential efficacy and safety of intravesical alpha therapy with the [^211^At]At-anti-CA-IX antibody (ATO-101™) in NMIBC treatment. Preclinical and clinical data demonstrate the promising therapeutic role of [^211^At]At-targeted alpha agents in NMIBC and the [^211^At]At-anti-CA-IX antibody (ATO-101™) could fulfill this role.

## 1. Introduction

In recent years, targeted radionuclide therapy has undergone significant clinical development [1]. Lutetium-177 has mostly replaced iodine-131, which has been used for several decades to label antibodies or peptides for therapeutic purposes. Recently, several therapeutic radiopharmaceuticals using lutetium-177 have been approved in the USA and the European Union [2,3], and a new generation of alpha-emitting radionuclides is now in active clinical development with promising preliminary results [4,5]. Among the potentially usable alpha-emitting radionuclides, astatine-211 (^211^At) is available from mid-energy cyclotrons [6]. To match up to the characteristics of astatine-211 (^211^At), the clinical target must be rapidly accessible because of its short physical half-life (7.2 h) and must be small because of the very short path of the alpha particles emitted, which is typically less than 100 µm [7]. A first clinical study was carried out in 2008 with an anti-tenascin antibody labeled with ^211^At with endocavitary instillation into a surgically created resection cavity of patients with recurrent malignant brain tumors [8]. More generally, endocavitary instillation enables rapid access to tumor targets.

Non-muscle-invasive bladder cancer (NMIBC) fits these characteristics. In fact, NMIBCs are either limited to the mucosa [9], such as pTa and urothelial carcinoma in situ (CIS), or invade the submucosal layer (usually high-grade pT1). Among NMIBCs, 70% are classified as pTa, 20% as pT1, and the remaining 10% as CIS lesions [10]. The proposed treatments are transurethral resection of the bladder (TURB) and intravesical administration of the bacillus Calmette–Guerin vaccine (BCG) or mitomycin, but recurrence rates are 50–70%. Iterative TURBs ± new bladder instillations may then be proposed depending on the NMIBC grade, but high-grade recurrence (pTa and pT1) remains an issue and radical cystectomy may be required prior to muscle invasion of the disease [11]. However, the therapeutic benefits of radical cystectomy are offset by a drastic reduction in quality of life. For this reason, new therapies (i.e., immunotherapies, immune checkpoint inhibition) that leave the bladder intact have been used in clinical trials in the instillation-resistant stage of NMIBC, with therapeutic efficacy in some patients [12]. Preclinical and clinical radioimmunotherapy, targeting epidermal growth factor receptor (EGFR), mainly expressed in CIS, with an antibody-radiolabeled with an alpha-emitting radionuclide (bismuth-213), has been proposed [13,14], but CIS accounts for only 10% of NMIBCs [10]. However, a good tolerance and encouraging efficacy justified the use of the intravesical route with short-lived alpha-emitting radionuclides.

Klatte et al. investigated CA-IX [15,16], which belongs to the group of membrane-associated carbonic anhydrase enzymes and is a member of the metalloenzyme family. The CA-IX antibody, known in the literature as girentuximab, targets the CA-IX antigen. CA-IX is not expressed in most healthy tissues, including normal urothelial tissues [17]. In contrast, expression is seen in 70–90% of NMIBCs but rarely in CIS [18]. CA-IX expression is associated with T-classification and NMIBC grade, with higher expression levels in Ta tumors than in invasive carcinomas, but also higher levels in grade 1–2 tumors than in grade 3 [16]. Consequently, alpha therapy using astatine-211-labeled girentuximab (trade-marked ATO-101™) can meet the prerequisites for a successful therapy in this indication, as has been shown with an actinium-225-labeled anti-CA-IX antibody in the context of clear cell renal cell carcinoma [19]. Finally, preclinical and clinical studies have revealed a BCG-mediated induction of innate immunity [20]. In similar ways, targeted alpha therapy was able to remodel the tumor microenvironment and improve the efficacy of immunotherapy [21,22].

Our study had two objectives: The first objective was to preclinically evaluate the efficacy of the [^211^At]At-anti-CA-IX antibody (ATO-101™), an astatine-211-labeled anti-CA-IX antibody, in a bladder cancer cell line and ATO-101™ biodistribution in healthy mice. Secondly, a clinical feasibility study (Pertinence, NCT04897763) was undertaken involving PET/CT imaging in six patients using [^89^Zr]Zr-girentuximab to demonstrate uptake and the absence of radioactivity diffusion beyond the bladder following intravesical instillation.

## 2. Materials and Methods

### 2.1. Radiochemistry

#### Labeling of Girentuximab with At-211 ([^211^At]At-anti-CA-IX Antibody (ATO-101™))

All reagents and solvents were obtained commercially and used without further purification unless otherwise noted. ^211^At was produced at the Arronax cyclotron facility using ^209^Bi(α, 2n)^211^At and recovered from the irradiated target in chloroform using a dry-distillation protocol [23]. Astatination of girentuximab was performed using a two-step process from a biaryliodonium salt precursor of N-succinimidyl-3-[^211^At]-astatobenzoate ([^211^At]SAB) and adapted from a previous work [24]. The [^211^At]SAB prosthetic group was then conjugated to a girentuximab antibody in borate buffer 0.3M pH 8.6 (5 mg/mL); after incubation for 30 min at room temperature, the radiolabeled antibody was then purified using a PD-10 size exclusion chromatography column (Sephadex G25, Cytiva, Saint-Germain-en-Laye, France) with saline solution (0.9% NaCl) as the eluent. Radiochemical purity was evaluated by iTLC-SG analysis (methanol as the eluent) and was always above 95%. These values were confirmed by HPLC analyses performed on an analytical system (Waters Alliance e2695, Saint-Quentin-en-Yvelines, France) using a size exclusion chromatography column (Superdex 200 10/300 GL, Saint-Quentin-en-Yvelines, France) with a flow rate of 0.5 mL.min-1 and an isocratic of phosphate-buffered saline. Finally, the immunoreactive fraction of [^211^At]-girentuximab was determined using magnetic beads (Pierce™ Magnetic Beads, Illkirch, France) conjugated with Human Carbonic Anhydrase IX/CA9 (Acro Biosystems, Newark, NJ, USA), which is recognized by the girentuximab antibody, according to the supplier’s protocol. Usually, an immunoreactive assay was performed using 0.1 pmol of labeled antibody that was incubated for 30 min at room temperature with magnetic beads. Then, using a magnetic rack, supernatants containing non-reactive antibodies and magnetic beads were collected separately and the radioactivity in each fraction was measured in a gamma counter. The immunoreactive fractions of the labeled antibody were always in the range of 75–81%.

### 2.2. Preclinical Study

#### 2.2.1. Biodistribution of [^211^At]At-anti-CA-IX Antibody (ATO-101™) in Healthy Mice

Nine female CD-1 mice, divided in groups of three, received about 1200 kBq of [^211^At]At-anti-CA-IX antibody (ATO-101™) into the bladder cavity via catheterization. Mice were water-deprived before instillation, with the bladder mechanically evacuated and a minimal volume (30 µL) instilled. Mice were sacrificed at 30 min, 1 h, and 4 h, respectively, and ^211^At activity was measured in various organs and tissues. The concentration of radioactivity was expressed as the percentage of the injected dose per gram of organ (%ID/g) and as the percentage of the ID for the whole organ.

#### 2.2.2. Toxicity Evaluation of [^211^At]At-anti-CA-IX Antibody (ATO-101™) in Healthy Mice

The aim was to assess the toxicity of the [^211^At]At-anti-CA-IX antibody (ATO-101™) after intravesical instillation in healthy mice, with an activity of 0.7 MBq considered high in the context of extrapolation to humans. Indeed, 0.7 MBq of [^211^At]At-anti-CA-IX antibody (ATO-101™) administered in 30 µL to mice is equivalent to 933 MBq in 40 mL in human patients in terms of volume activity (23.3 MBq/mL). The [^211^At]At-anti-CA-IX antibody (ATO-101™) was intravesically instilled in 5 female CD-1 mice through a catheter who were housed for 15 days before sacrifice. Bladders and kidneys were collected and then fixed in paraformaldehyde to allow for histological examination.

#### 2.2.3. Measurement of Affinity Constant of [^211^At]At-anti-CA-IX Antibody in RT-112 Cells

Human urinary bladder transitional cell carcinoma RT-112 was provided by the European Collection of Authenticated Cell Cultures (ECACC reference: RT112/84). The cells were grown in Eagle’s Minimum Essential Medium (EMEM) supplemented with 10% Fetal Bovine Serum (FBS) and 2 mM Glutamine at 37 °C and 5% CO_2_. A suspension of RT-112 cells at 20 × 10^6^ cells/mL was prepared in PBS 1X pH 7.4 containing 0.5% (*w*/*v*) BSA.

For total binding determination, a serial dilution of [^211^At]At-anti-CA-IX antibody solution was performed in sample buffer covering the concentration range from 25 nM to 0.10 nM. For nonspecific binding determination, a solution containing 40 nM of [^211^At]At-anti-CA-IX antibody with 100-fold molar excess of unlabeled antibody, 4 µM, was prepared in sample buffer; then, a 4-fold dilution series in PBS/BSA 0.5% was generated including 5 dilutions.

For the saturation binding assay, RT-112 cells (1 × 10^6^ cells in 50 µL PBS/BSA 0.5%) were incubated in duplicate with increasing concentrations of radiolabeled antibody solution in a final volume of 150 µL for 90 min at room temperature under mixing. Following incubation, reaction mixtures were overlaid onto 200 µL of a dibutylphtalate oil cushion and centrifuged in microfuge tubes at 12,000 rpm for 3 min. Tubes were frozen in liquid nitrogen, and the tips of the tubes containing the cell pellets were cut off for determination of radioactivity using a gamma counter. The affinity constant of the [^211^At]At-anti-CA-IX antibody in RT112 cells and the maximum binding sites per cell were determined by Prism software version 10.3.1.

#### 2.2.4. Labeling of Girentuximab with Lutetium-177

The girentuximab antibody was conjugated with *p*-SCN-DOTA at a 1:7 molar ratio. After conjugation, the immunoconjugate was purified by ultracentrifugation using Amicon-30 kDa to remove any unconjugated chelating agent in the solution. Chemical purity was assessed by SE-HPLC and SDS-PAGE, and immunoconjugate DOTA-girentuximab concentration was determined by absorptivity at 280 nm. An average of 3 DOTA chelators per girentuximab antibody were identified. The final solution of DOTA-girentuximab, formulated in PBS 1X, pH 7.4, at 5.18 mg/mL, was stored at 4 °C until labeling. DOTA-girentuximab was radiolabeled with lutetium-177 to reach 1000 MBq/mg final specific activity and 800 MBq/mL final concentration. Radiolabeling of DOTA-girentuximab was performed using [^177^Lu]LuCl3 (EndolucinBeta™/ITG, Garching, Germany). Radiochemical purity was determined by SE-HPLC, and ITLC was revealed by autoradiography. To prevent degradation and maintain high radiopurity, [177Lu]Lu-girentuximab was formulated in 0.03 M sodium phosphate, 200 μg/mL BSA, and 80 μg/mL sodium ascorbate.

#### 2.2.5. Analysis of Compared Cell Toxicity Using [^177^Lu]Lu-Girentuximab and [^211^At]At-anti-CA-IX Antibody (ATO-101™)

In vitro cytotoxicity of the [^211^At]At-anti-CA-IX antibody (ATO-101™) was assessed in comparison to [177Lu]Lu-girentuximab, in the CA-IX-positive cell line RT112 following 2 h exposure to various radioactive concentrations. The viability of cells was assessed at Day 2 and Day 5, using the colorimetric MTS assay.

#### 2.2.6. Clinical Proof of Concept of Intravesical PET Imaging in Patients: PERTINENCE Study Procedure Using [^89^Zr]Zr-Girentuximab

To evaluate the tumor targeting of CA-IX by Zirconium-89-labeled anti-CA-IX antibody administrated intravesically to transitional cell carcinoma (TCC) of the bladder in patients, a clinical study was designed with a Zirconium-89-labeled anti-CA-IX antibody for PET/CT imaging. This pilot prospective study aimed, firstly, to ensure intravesical radioactivity confinement by PET/CT imaging after [^89^Zr]Zr-girentuximab instillation; then, PET/CT bladder pattern was compared to the degree of CA-IX expression (percentage and intensity of tumor cell expression), evaluated by immunohistochemistry (IHC).

Ethical approval was obtained from the Ile de France III Review Board (21.05.10.47712). All patients signed a written informed consent form prior to participation in this study (2021-001709-61).

Six patients (four men and two women), aged between 71 and 80 years, with biopsy-proven TCC (pTaG3 for all of them), were enrolled between December 2021 and August 2022.

#### 2.2.7. PET/CT Imaging

Patients had one intravesical instillation of 37 ± 10% MBq of [^89^Zr]Zr-girentuximab (10 mg antibody mass dose) and retained urine for 2 h before emptying their bladders. Then, four PET/CT VISION 450 (Siemens, Erlangen, Germany) scans were performed, three with one step on the pelvis (hours + 2 after instillation on Day 0 then on Day 1 and Day 2) and one whole-body scan from the skull to mid-thigh at H + 4 D1 to observe intravesical radioactivity evolution over time. For all acquisitions, a 10 min acquisition time per bed position was used. The gold standard was determined by a second-look cystoscopy with a few TURBs if there were bladder foci detected on [^89^Zr]Zr-girentuximab PET/CT.

#### 2.2.8. Quantitative ^89^Zr Activity Analysis in Blood

The ^89^Zr activity in patient blood samples (3 blood vials, 1 mL each) was determined before injection (background noise, Bkg) and on Day 1 after injection by counting the [^89^Zr]Zr-girentuximab activity in blood in a calibrated gamma counter (Wizard 2480, PerkinElmer, Villepinte, France) with a 61.4% counter efficiency.

#### 2.2.9. Immunohistochemistry for CA-IX Expression

Immunohistochemistry (IHC) was performed with an anti-CA-IX antibody (clone EP161, Cell Marque, dilution 1:100). Staining was evaluated with semi-quantitative analysis (percentage and intensity of tumor cell expression) and [^89^Zr]Zr-girentuximab PET/CT bladder pattern compared to the degree of CA-IX expression evaluated by IHC.

#### 2.2.10. Dosimetry Estimation

The absorbed dose estimation of healthy tissue given per unit of activity administered, in mGy/MBq, was based on the MIRD formalism and the calculation of cumulative activity for ^211^At in a single source volume, in this case, the instilled bladder, with the bladder wall as the single target volume. The residence time considered for healthy tissue was two hours, corresponding to the instillation duration since the radiopharmaceutical was washed out. Calculations were based on a reference geometry, a human female and/or male mathematical model defined in the ICRP 89 [25], and involved a specific absorbed fraction (SAF) previously calculated by simulation on the models in question and tabulated in the ICRP 133 [26].

Calculations were implemented with IDAC-Dose 2.1 software [27]. The contribution of ^211^Po as well as ^207^Bi (albeit of negligible contribution) have been added to the calculation, as IDAC-Dose 2.1 does not consider daughter elements.

Relative absorbed dose contributions were calculated for alpha particles, electrons, and photons. In order to take into account the relative biological effectiveness of alpha particles for deterministic effects, we applied a weighting factor equal to 5 to the calculation of the absorbed dose of this contribution (in mSv/MBq).

**Figure 1:**
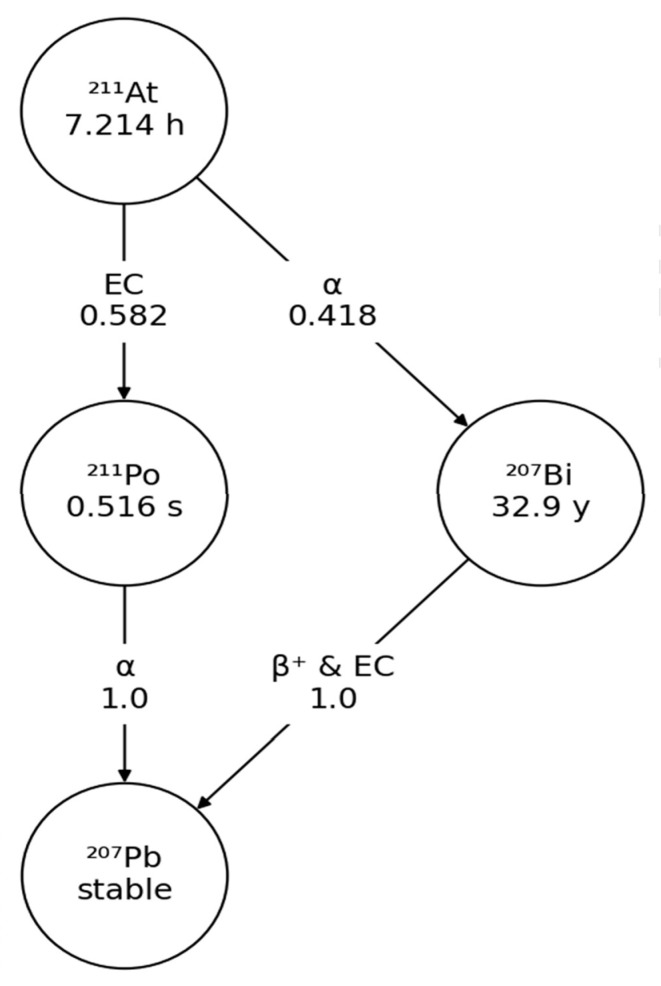
Decay scheme of astatine-211. Equivalent of one alpha particle is emitted within disintegration of astatine-211. Alpha particle emitted by astatine-211 in a 41.8% ratio with bismuth-207 has a kinetic energy of 5869.5 keV and a maximum path in water equal to 47.4 µm. Alpha particle emitted by polonium-211 in a 58.2% ratio with stable lead-207 has a kinetic energy of 7594.5 keV and a corresponding maximum path in water equal to 68.7 µm.

The calculation of absorbed dose considered the decay chain of astatine-211, namely polonium-211 and bismuth-207 (Figure 1).

We used the SRIM-2013 software to determine the maximum path of alpha particles in water emitted in the decay chain of astatine-211 [28].

## 3. Results

### 3.1. Measurement of Affinity Constant of [^211^At]At-anti-CA-IX Antibody in RT112 Cells

The binding characteristics of the [^211^At]At-anti-CA-IX antibody for RT112 cells showed a high binding affinity of the [^211^At]At-anti-CA-IX antibody for CA-IX expressed by RT112 cells with KD higher than 0.8 nM and a number of binding sites near 10,000 sites per cell, fully compatible with therapeutic targeting described below by the in vitro study.

### 3.2. Compared Cytotoxicity in RT112 Cells

**Figure 2:**
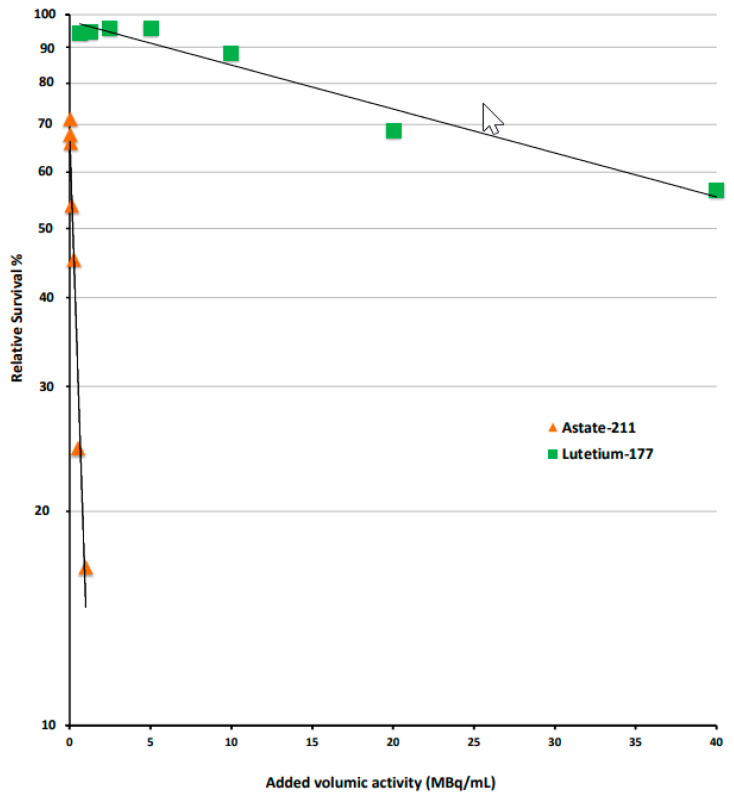
Compared cytotoxic effect of girentuximab labeled with either 177Lu (green square) and 211At (orange triangle) on RT112 cells at D5 after exposure.

Survival curves of RT112 cells obtained after exposure at different concentrations of α-emitting [^211^At]At-anti-CA-IX antibody (ATO-101™) and β-emitting [177Lu]Lu-girentuximab on RT112 cell survival are presented in Figure 2. Significant differences were observed in the slopes of the survival curves. Exposure of RT112 cells to 1 MBq/mL of [^211^At]At-anti-CA-IX antibody (ATO-101™) led to 85% cell death, whereas exposure to a 40-times-greater concentration, i.e., 40 MBq/mL of [^177^Lu]Lu-girentuximab, led to a very modest cytotoxic effect estimated at about only 45% cell death.

### 3.3. Biodistribution of [^211^At]At-anti-CA-IX Antibody (ATO-101™) in Healthy Mice

**Figure 3:**
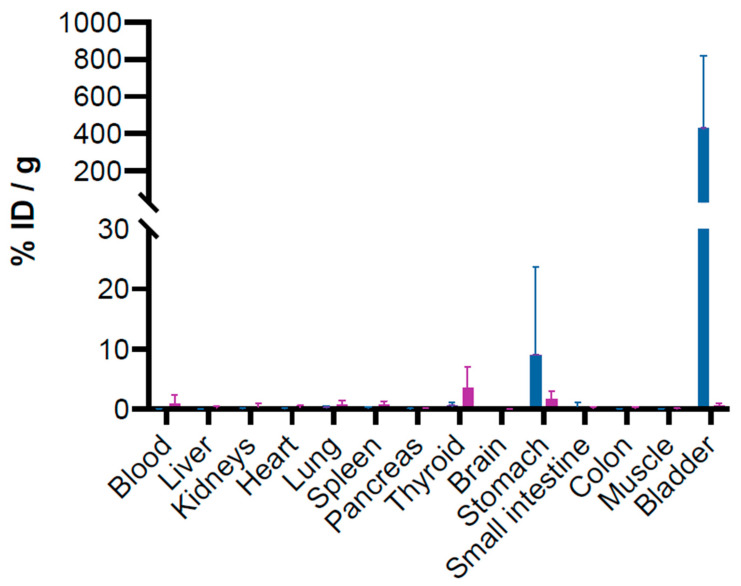
Biodistribution of 1200 kBq of [^211^At]At-anti-CA-IX antibody (ATO-101™) at 30 (blue square), 60 (yellow square), and 240 min (violet square) after intravesical instillation in bladders of tumor-free mice. Biodistribution data of [^211^At]At-anti-CA-IX antibody (ATO-101™). Three animals were sacrificed at each time point. Data are expressed as the percentages of injected dose per gram of tissue (%ID/g). Means and SDs are depicted.

As expected, the biodistribution study in healthy mice showed low radioactive uptake in systemic organs and tissues (<1% injected dose/gram) at 1 h, except in the bladder, which is the consequence of previous intravesical instillation. Hence, radioactive accumulation remained elevated despite the bladders being rinsed and emptied according to protocol. Four hours after emptying, a slight increase in activity was observed in some tissues such as the blood, lung, spleen, and, mainly, stomach. Activity in the stomach is postulated to be due to ingestion related to animal self-grooming after instillation rather than a biodistribution effect (Figure 3).

Data obtained from Mouse 2 (group 30 min) largely corroborate this explanation. The low level of activity in the bladder (1.38%ID/g) compared to the two others (518.80%ID/g and 776.50%ID/g) can probably be attributed to a fast and complete emptying of the bladder due to inconvenience caused by the urinary catheter and leading to the increase in activity in the stomach (25.87%ID/g compared to 0.49%ID/g and 0.33%ID/g for Mice 1 and 3).

### 3.4. Cytotoxicity Evaluation in Healthy Mice

Histological examination of the bladder mucosa showed no microscopic abnormal observations which could be attributed to the experimental procedure. The extrapolation of these data in healthy mice (0.7 MBq in 30 μL volume of [^211^At]At-anti-CA-IX antibody (ATO-101™) dosed intravesically) corresponds to an activity of 933 MBq in 40 mL of [^211^At]At-anti-CA-IX antibody (ATO-101™) intravesically instilled in human bladder.

### 3.5. Clinical Proof of Concept of Intravesical Therapy in Patients: PERTINENCE Study Procedure Using [^89^Zr]Zr-Girentuximab

**Figure 4:**
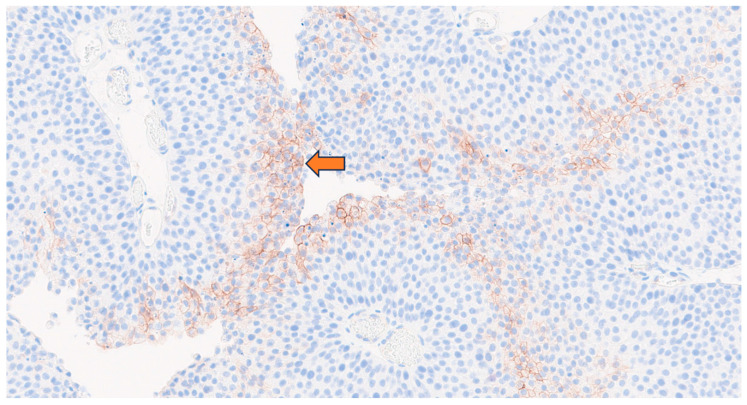
CA-IX staining is observed on papillary structure luminal surface (arrow) in direct contact with bladder cavity.

**Figure 5:**
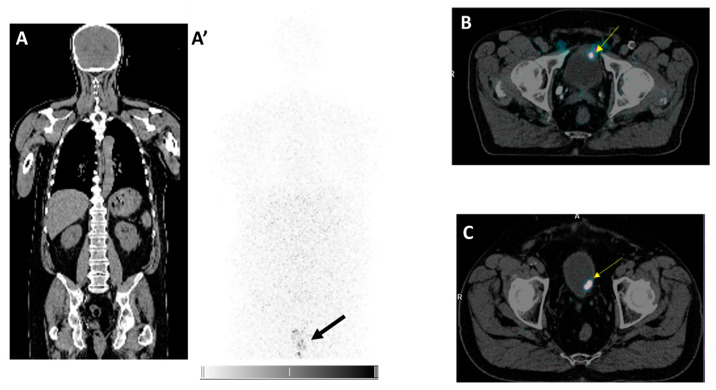
(**A**) CT coronal and (**A′**) [^89^Zr]Zr-TLX250 PET of the whole body with no radioactivity in the body, except for a bladder residue (2 h after instillation and urination) (bold arrow); (**B**) Day 1, uptake focus (yellow arrow) on fusion axial [^89^Zr]Zr-TLX250 PET-CT corresponding to TURB-controlled CA-IX IHC tumor recurrence in patient 1 (detailed in Table 1); (**C**) Day 1, uptake focus (yellow arrow) on fusion axial [^89^Zr]Zr-TLX250 PET-CT showed TURB-controlled CA-IX IHC scar in patient 2 (detailed in Table 1).

Table 1 showed patient characteristics and immunohistochemistry results of the various biopsy sample collections. Four patients were treated previously with BCG instillations alone (one to two complete cycles ± maintenance) and two patients also received one cycle of mitomycin instillations. All patients had NMIBC pTaG3 at study inclusion and all tissue samples expressed CA-IX except for one patient. CA-IX was expressed at the membrane of tumor cells, in less than or equal to 20% of cells, with moderate intensity (2+). The immunostaining was preferentially observed on luminal papillary surfaces along the lumen as a band (Figure 4). The median time between the last TURB and [^89^Zr]Zr-girentuximab injection was 8 weeks (range, 4.5–15). In the first patient, the delay between TURB and [^89^Zr]Zr-girentuximab imaging was 4.5 weeks, and pelvic imaging showed radiopharmaceutical leakage around resected bladder areas. The time between TURB and imaging was prolonged in the next patients, so that at 6 weeks post-TURB, the bladder was leak-free again. [^89^Zr]Zr-girentuximab PET/CT pelvis acquisitions have shown, in two out of six patients, the presence of ureteral reflux. [^89^Zr]Zr-girentuximab PET/CT of the whole body showed no extravesical leakage (Figure 5A,A′), except for in one patient whose urethra had been injured during the insertion of the urinary catheter. This imaging aspect was confirmed by the quantitative analysis of the blood samples taken from the patients: the blood results of five patients corresponded to the sample taken before the radiopharmaceutical injection, whereas in the injured patient, 151 Bq/mL of blood was detected, which corresponds to 2.1% of the injected activity (conversion factor 36.8 cpm/Bq), if we relate this activity to the patient’s blood volume.

In three out of six patients, wall bladder uptake spots, seen on [^89^Zr]Zr-girentuximab PET/CT pelvis acquisitions, were investigated by TURB post-imaging. For one patient, this corresponded to an early recurrence of NMIBC pTaG3 with similar or higher CA-IX expression, but always with moderate intensity (2+) (Figure 5B). For the other two patients, it corresponded to post-TURB inflammatory scarring reaction with positive IHC for one of them (Figure 5C). During the injection and imaging process, no adverse event or contamination event occurred, and no measurable worker exposure was observed.

### 3.6. Dosimetry Estimation

Dosimetry results showed an average dose per unit of activity to the complete bladder wall from the urinary content of 0.22 mGy/MBq, resulting from 0.20 mGy/MBq plus 2.24 × 10^−4^ mGy/MBq plus 1.53 × 10^−2^ mGy/MBq for alpha particles, electrons, and gammas, respectively. This highest normalized absorbed dose is obtained with the female mathematical model due to the smaller volume of the target, in this case, the bladder wall, compared to the male model. Assuming an RBE of 5 for alpha particles for deterministic effects, the equivalent absorbed dose from the different emission types were estimated as 1.01 mSv/MBq, 2.24 × 10^−4^ mSv/MBq, and 1.53 × 10^−2^ mSv/MBq for alpha particles, electrons, and gammas, respectively, meaning the alpha particles contributed most to energy deposition. The total equivalent absorbed dose was estimated in the ICRP model as 1.02 mSv/MBq and 6.19 × 10^−3^ mSv/MBq/cm^2^ (Table 2). The maximum path of the two alpha particles emitted along the decay chain, calculated with SRIM-2013 [28], was 47.4 µm and 67.8 µm for the 5.9 MeV and 7.6 MeV emission energies, respectively. In addition, the geometry of the volume source and target volume are of major importance. The urinary bladder content volume considered in the ICPR model is 200 mL, while the instilled volume in our clinical context is 40 mL. This result in a three-factor impact on the concentration activity on the surface with the potential influence of alpha vicinity to the bladder wall cells. In a first approximation, one may consider a pejorative absorbed dose per unit of activity in the bladder wall of 0.65 mGy/MBq. Therefore, the maximum instilled activity planned in our study is 444 MBq, yielding a maximum estimated absorbed dose of 0.29 Sv.

## 4. Discussion

This preclinical study in an in vitro and in vivo bladder cancer model demonstrated the relevance of targeting carbonic anhydrase with an anti-CA-IX antibody (girentuximab). The intravesical radiopharmaceutical delivery approach clearly demonstrates good targeting of bladder cancer cells without toxicity. The major conclusions of the clinical proof-of-concept feasibility study are that the elimination of asymptomatic vesicoureteral reflux after all TURBs is a prerequisite for treatment, that a delay of at least 8 weeks between TURB and treatment to allow the bladder wall to repair, ensuring bladder tightness, is also imperative, and, finally, that post-TURB scars persist > 8 weeks but this persistence of CA-IX expression could be favorable for the recommended treatment.

The high cytotoxicity of alpha particles due to their linear energy transfer opens up interesting therapeutic prospects for small therapeutic targets and broadens the indications of targeted radiotherapy, which is currently in advanced development with alpha-emitting radionuclides. The difference was illustrated in the preclinical part of this study by the comparative cell survival observed with the anti-CA-IX (girentuximab) antibody labeled with lutetium-177 and astatine-211. The [^211^At]At-anti-CA-IX antibody (ATO-101™) showed significantly greater efficacy than [^177^Lu]Lu-DOTA-CA-IX (girentuximab) in terms of cytotoxicity. Although the two mechanisms of action are significantly different, exposure of RT112 cells to 1 MBq/mL of ATO-101™ resulted in 85% cell death, whereas exposure to a 40-fold-higher concentration, i.e., 40 MBq/mL of [177Lu]Lu-girentuximab, resulted in a very modest cytotoxic effect, estimated at only 45% cell death. This therapeutic efficacy of alpha particles must be weighed against their very short path length of less than 0.1 mm, which limits their action to very small tumor targets, ideally clusters of residual tumor cells proximal to the dose delivery site. In this context, the indication of non-muscle-invasive bladder cancer corresponds to the characteristics of the anti-CA-IX antibody girentuximab and ^211^At. Papillary bladder tumors are located on the luminal surface of the bladder mucosa. After resection, they persist as microscopic tumor residues. Given the lack of established therapy after failure of BCG treatment, these tumor residues represent a preferred therapeutic target for the [^211^At]At-anti-CA-IX antibody (ATO-101™). An a priori favorable advantage of endocavitary alpha-immunotherapy, which needs to be confirmed in a phase I study, is its limited local and systemic toxicity. Indeed, no toxicity was reported in the pilot study conducted with a bismuth-213-labeled anti-EGFR antibody in 12 patients [13] and in the present preclinical study conducted with the [^211^At]At-anti-CA-IX antibody (ATO-101™) in healthy mice.

The intravesical administration of the [^211^At]At-anti-CA-IX antibody (ATO-101™), targeting bladder tumor cells, is anticipated to minimize systemic toxicity. This has been supported by biodistribution studies, which revealed exceptional retention of the radiopharmaceutical within the bladder, as demonstrated by the use of a bismuth-213-labeled anti-EGFR antibody by Reingard Senekowitsch-Schmidtke’s team [14]. In addition, no histological abnormalities of the bladder mucosa of healthy mice were reported after intravesical instillation of 700 kBq. In human clinical terms, this corresponds to an activity of 933 MBq more than the maximum activity of 821 MBq already tested in a phase I study with the bismuth 213-labeled anti-EGFR antibody [13]. Moreover, recent advances in radiobiology and immunology suggest that irradiation with alpha particles adds an immune response component to the direct tumoricidal effect [21,29].

In the proof-of-concept PERTINENCE study, our cohort consisted of patients with pTaG3 NMIBC. They were therefore long-term candidates for radical cystectomy due to a 17% disease risk of progression to an infiltrative tumor [11]. In all but one patient, CA-IX expression was detected on the pre-imaging TURB specimen. This ratio is consistent with that reported in the literature, with CA-IX expression in 70–90% of NMIBCs [18].

Pelvic [^89^Zr]Zr-girentuximab PET/CT revealed asymptomatic vesicoureteral reflux in two of our patients. This appearance was the result of multiple TURBs performed on the patients, particularly in the areas of the ureteral orifices. Kisbenedek et al. reported incidence of asymptomatic unilateral vesicoureteral reflux after 30% TURB and, exceptionally, bilateral reflux (3%) [30]. In order not to miss a vesicoureteral reflux, we propose to perform a [^99m^Tc]Tc-MAG3 scintigraphy prior to the future alpha-emitting therapy trial, as described in the literature [31].

One of the key determinants at the time of TURB is whether detrusor muscle was obtained. All the international guidelines highlighted the importance of fully resecting all of the visible tumor and obtaining muscle at the time of TURB [32]. However, the bladder wall needs to repair itself after TURB. The pelvic [^89^Zr]Zr-girentuximab PET/CT showed leakage of the radiopharmaceutical into the perivesical fat in TURB areas in the first two patients who had a short delay between TURB and PET/CT. With regard to alpha-emitting therapy, it will be important to allow sufficient time for the bladder wall to become impermeable again, and 8 weeks seemed to be an appropriate time frame.

The literature showed that CA-IX is involved in the skin healing process. Indeed, the expression of mRNAs encoding CA isoform IX is increased (~4 times) during the wound hypoxic period (days 2–5) and cells expressing CA-IX form a band-like structure beneath the migrating epidermis [33]. However, there is no mention of a similar observation in any tissue other than skin. We have shown the persistence of CA-IX expression on an inflammatory and scarred non-tumor bladder wall 6 weeks after TURB, well beyond the 2–5 days described for skin healing.

Finally, in five out of six patients, it was shown that it was possible to administer a radiopharmaceutical intravesically without significant additional radiation to personnel or contamination. As already described in the study by Autenrieth et al., the radiopharmaceutical was retained in the bladder for 120 min without any particular problems, despite the fact that the bladder was treated several times before [13]. We would like to emphasize that even the slightest vascular invasion cannot be tolerated because it leads to diffusion of the radiopharmaceutical throughout the body, as was the case in one of our patients. With regard to the results of the dosimetric approach, it is important to have in mind that the main characteristic of astatine-211 from a dosimetry point of view is the dominant contribution of alpha particles in the energy deposition of the absorbed dose. Due to the finite short path of these particles (50 to 70 µm), the geometry underlaying the source and target volume is a major topic. The bladder wall is a stack of cell layers of which the external one (to the right of the bladder) consists of non-active cells (umbrella cells) which are not sensitive to ionizing radiation. The radiosensitive basal layer cells lay from 60 to 120 µm from the surface, depending on the bladder filling volume, meaning that, depending on the conformation of the bladder, only a few alpha particles, or even none, would hit basal cells. The estimated absorbed dose by the model is a mean within the target volume and does not consider the way that energy is deposited. Based on patients with the highest predicted uptake of 0.65 mGy/MBq (female model due to the lower target mass and considering a volume effect from the model), even the highest proposed dose of 444 MBq [^211^At]At-anti-CA-IX antibody (ATO-101™) would indicate an estimated dose of just 0.29 Gy in the healthy bladder wall. This figure was two orders of magnitude lower than the tolerance dose of 40 Gy defined by Meredith et al. [34]. This is in line with the results obtained in Autenrieth’s study, although the relative contributions of alpha, beta, and gamma particles to the dose absorbed in the tissues between astatine-211 and bismuth-213 explain the lower absorbed dose in healthy tissues in the PERTINENCE study. Regarding the tumor cells, which, in contrast, are superficial and easily targeted by the [^211^At]At-anti-CA-IX antibody (ATO-101™), one may hypothesize, based on the same argument, that the local energy deposition will be high due to alpha particles, contributing to a high efficacy. Nevertheless, extensive studies with more sophisticated tools are required to figure out the calculation dose to the tumor lesions, based on the number of binding sites per cell.

The clinical value of alpha-immunotherapy is promising based on the first clinical results [8] and should rapidly be confirmed in various indications and with several alpha-particle-emitting radionuclides. Currently, two phase I clinical studies using ^211^At have been completed, and five more are ongoing [35]. The main problem to be solved through such clinical development with ^211^At is its availability for large-scale distribution and use. ^211^At has the advantage of being able to be produced by a medium-energy cyclotron, but its short physical half-life of 7.2 h means that a distribution network covering the entire country needs to be organized.

## 5. Conclusions

Collectively, preclinical and clinical data demonstrate the promising role of ^211^At-targeted alpha agents and support a phase I FIH study with the [^211^At]At-anti-CA-IX antibody (ATO-101™) for patients after BCG therapy failure. A significant unmet medical need exists for patients after BCG therapy failure which the [^211^At]At-anti-CA-IX antibody (ATO-101™) could fulfill.

## Figures and Tables

**Table 1:** Patient characteristics and immunohistochemistry results (CA-IX-EP161 Cell Marque).

Patient	Sex	Age (Years)	Initial TCC Diagnosis	Treatments Received	TURB Pre-Imaging	89Zr-TLX250 Imaging	TURB Post-Imaging	FFPE Samples	Biopsy Results	CA-IX—EP161 Cell Marque	
										Positive Cells %	Intensity (1+, 2+, 3+)
1	M	80	Oct 2020	BCG × 6: Apr–May 2021	Nov 2021	Dec 2021	Feb 2022	TURB pre-imaging	Non-invasive papillary urothelial cancer, pTaG3	10	2+
								*Biopsy post-imaging*	*Non-invasive papillary urothelial cancer, pTaG3*	*20*	*2+*
								*Biopsy post-imaging*	*Non-invasive papillary urothelial cancer, pTaG3*	*10*	*2+*
2	M	72	Jun 2021	BCG × 6: Aug–Sept 2021	Dec 2021	Jan 2022	Feb 2022	TURB pre-imaging	Non-invasive papillary urothelial cancer, pTaG3	3	2+
								*Biopsy post-imaging*	*Inflammatory bladder wall without tumor*	*0*	*Not applicable*
3	F	71	May 2004	BCG × 6: Jul–Aug 2005 Mitomycin x 6: Nov–Dec 2006 BCG × 6: Nov–Dec 2005 BCG × 3: Apr 2007 + Oct 2007	Nov 2021	Feb 2022	-	TURB pre-imaging	Non-invasive papillary urothelial cancer, pTaG3	2	2+
4	M	69	Feb 2019	Mitomycin × 6: Apr–Jun 2019 BCG × 6: Aug–Sept 2000	Jan 2022	Mar 2022	-	TURB pre-imaging	Non-invasive papillary urothelial cancer, pTaG3	0	Not applicable
5	M	73	Nov 2016	BCG × 6: Apr–May 2016 BCG × 6: Mar–Apr 2019 BCG × 3: Aug 2020+ Janv 2021	Jan 2022	Mar 2022	-	TURB pre-imaging	Non-invasive papillary urothelial cancer, pTaG3	1	2+
6	F	77	Aug 2021	BCG × 6: Nov–Dec 2021	Jul 2022	Aug 2022	Sept 2022	TURB pre-imaging	Non-invasive papillary urothelial cancer, pTaG3	5	2+
								*Biopsy post-imaging*	*Inflammatory and scarred bladder wall without tumor*	*10*	*2+*

TCC: transitional cell carcinoma of the bladder; TURB: transurethral resection of the bladder; FFPE: formalin-fixed, paraffin-embedded.

**Table 2:** IDAC-Dose 2.1 software-estimated absorbed dose equivalent to the bladder wall as a function of the isotope involved in the decay chain of ^211^At. ^207^Bi-absorbed dose contribution is negligible. A weighting factor of 5 has been considered for the alpha particles, and 1 for electrons and photons.

Radionuclide	Residence [h] Time	Absorbed Dose per Unit of Activity Administered [mSv/MBq]	
		Male	Female
^211^At	1.820	0.158	0.195
^211^Po	1.819	0.662	0.827
Total		0.820	1.022

## Data Availability

The datasets generated during and/or analyzed during the current study are available from the corresponding author on reasonable request.
